# Zinc Protoporphyrin Upregulates Heme Oxygenase-1 in PC-3 Cells via the Stress Response Pathway

**DOI:** 10.1155/2013/162094

**Published:** 2013-02-17

**Authors:** Simon C. M. Kwok

**Affiliations:** ORTD, Albert Einstein Medical Center, 5501 Old York Road, Korman 214, Philadelphia, PA 19141-3098, USA

## Abstract

Zinc protoporphyrin IX (ZnPP), a naturally occurring molecule formed in iron deficiency or lead poisoning, is a potent competitive inhibitor of heme oxygenase-1 (HO-1). It also regulates expression of HO-1 at the transcriptional level. However, the effect of ZnPP on HO-1 expression is controversial. It was shown to induce HO-1 expression in some cells, but suppress it in others. The objective of this study is to investigate the effect of ZnPP on HO-1 expression in prostate cancer PC-3 cells. Incubation of PC-3 cells with 10 **μ**M ZnPP for 4 h showed only a slight induction of HO-1 mRNA and protein, but the induction was high after 16 h and was maintained through 48 h of incubation. Of all the known responsive elements in the HO-1 promoter, ZnPP activated mainly the stress response elements. Of the various protein kinase inhibitors and antioxidant tested, only Ro 31-8220 abrogated ZnPP-induced HO-1 expression, suggesting that activation of HO-1 gene by ZnPP may involve protein kinase C (PKC). The involvement of PKC **α**, **β**, **δ**, **η**, **θ**, and **ζ** isoforms was ruled out by the use of specific inhibitors. The isoform of PKC involved and participation of other transcription factors remain to be studied.

## 1. Introduction

Heme oxygenase-1 (HO-1), also known as heat shock protein 32 (Hsp32), is an inducible enzyme that catalyzes the breakdown of heme, producing carbon monoxide, iron, and biliverdin. It is known to be a cytoprotective enzyme against oxidative stress [1]. It is often upregulated in tumor tissues, and its inhibition is considered as a means of sensitizing the tumors to anticancer drugs [2]. Although HO-1 expression is increased in malignant prostate tissues [3], its expression in prostate cancer cell line, PC-3, is low [4]. Induction of HO-1 expression by hemin in PC-3 cells resulted in decreased cell proliferation and migration [4]. Overexpression of HO-1 also led to nuclear location [5] and was associated with downregulation of matrix metalloprotease 9 (MMP9), which plays an important role in tumor cell invasion and angiogenesis [4]. The real function of HO-1 in tumor cells remains to be studied.

HO-1 expression can be induced by many inducers, and many regulatory pathways have been proposed [6, 7]. A number of antioxidant response element (ARE)-like motifs are present in the promoter of HO-1 gene. Six of these sites were found as clusters at E1 (−3928 bp) and E2 (−9069 bp) regions of the human HO-1 promoter; they are termed StRE1 through StRE6 [6]. Besides these StRE sites, other response elements, such as HSE [8], SREBP binding site [9], and an intronic SP1 enhancer [10] have also been reported to be present in HO-1 promoter. In addition, an Egr-1 binding site in mouse HO-1 promoter that is inducible by zinc protoporphyrin IX (ZnPP) has also been reported [11]. 

ZnPP, a naturally occurring molecule formed in iron deficiency or lead poisoning, is a potent competitive inhibitor of HO-1. Inhibition of HO-1 by ZnPP led to suppression of tumor cell growth [12] and ZnPP has been suggested to be a useful agent for antitumor therapy [13]. However, ZnPP has also been shown to regulate expression of HO-1 at the transcriptional level, and the effect of ZnPP on HO-1 expression is controversial. For example, it is shown to induce HO-1 expression in hamster fibroblast (HA-1) cells [11] but not in Neuro-2A mouse neuroblastoma cells and primary cultures of rat cortical neurons [14]. In fact, it even suppressed the induction of HO-1 by statins or lipopolysaccharide [14]. In our earlier study, ZnPP was found to induce HO-1 expression in human prostate adenocarcinoma PC-3 and breast adenocarcinoma MCF-7 cells [15]. It is a much stronger inducer of HO-1 than atorvastatin, one of the statins. In this study, we used prostate cancer PC-3 cells to investigate the mechanism of action of ZnPP. We herein report that ZnPP upregulates HO-1 in PC-3 cells via the antioxidant response pathway.

## 2. Materials and Methods

### 2.1. Reagents

N-acetyl cysteine (NAC) was product of Sigma-Aldrich (St. Louis, MO, USA). ZnPP and protein kinase inhibitors were purchased through EMD Chemicals Inc. (Gibbstown, NJ, USA). Antibodies against human *β*-actin and HO-1 were purchased from Cell Signaling Technology (Danvers, MA, USA) and Enzo Life Sciences (Plymouth Meeting, PA, USA), respectively. Antibodies against Keap1 and phospho-Nrf2 (pS40) were products of Proteintech Group, Inc. (Chicago, IL, USA) and Epitomics, Inc. (Burlingame, CA, USA), respectively. Antibodies against Bach1 were purchased from both Poteintech Group, Inc. and Epitomics, Inc.

### 2.2. Cell Line and Cell Culture

Human prostate adenocarcinoma PC-3 cell line was purchased from the American Type Culture Collection (Manassas, VA, USA). These cells were maintained as monolayer cultures in DMEM/F12 medium (Invitrogen, Carlsbad, CA, USA) supplemented with 10% fetal bovine serum, 100 units/mL of penicillin, 100 *μ*g/mL of streptomycin, and 0.25 *μ*g/mL of amphotericin B (complete medium) and were kept at 37°C in a humidified atmosphere containing 5% CO_2_.

### 2.3. Construction of Reporter Plasmids

The enhancer-luciferase reporter plasmids were constructed by inserting sequences of various synthetic response elements into the filled-in *Nhe*I*/Bgl*II sites of pGL3-promoter vector (Promega, Madison, WI, USA) or *Eco*RV site of pGL4-promoter vector via blunt-end ligation as described in our earlier study [15]. Internal control plasmid, pGL4.74[hRluc/TK], was purchased from Promega (Madison, WI, USA). 

### 2.4. RT-PCR and qPCR

PC-3 cells were grown to 80% confluence in T25 flasks and treated with 10 *μ*M ZnPP or equal amount of DMSO (vehicle) for various time intervals up to 48 h. Total RNA was extracted using NucleoSpin Nucleic Acid Purification Kits (Clontech, Palo Alto, CA, USA). First-strand cDNA was synthesized from 5 *μ*g of total RNA using ThermoScript (Invitrogen, Carlsbad, CA, USA) in a volume of 20 *μ*L. PCR was done for 30 cycles (denaturation at 94°C for 30 sec, annealing at 59°C for 30 sec, and extension at 72°C for 60 sec) using 1 *μ*L of the first-strand cDNA, 10 pmol of gene specific primers and 2.5 units of JumpStart Taq DNA polymerase (Sigma-Aldrich, St. Louis, MO) in a volume of 50 *μ*L. Primers for *β*-actin: 5′-CCTCGCCTTTGCCGATCC-3′ and 5′-GGATCTTCATGAGGTAGTCAGTC-3′. Primers for HO-1: 5′-AGAAGAGCTGCACCGCAAGG-3′ and 5′-CCTCTGAAGTTTAGGCCATTGC-3′. 

Real-time PCR (RT-qPCR) was performed with StepOne real-time PCR system (Applied Biosystems, Foster City, CA, USA) using TaqMan Gene Expression Master Mix and ready-made human HMOX1 (Hs 01110250_m1), ACTB (Hs 99999903_m1), NQO1 (Hs00168547_m1), GSTP1 (Hs00943351_g1), BACH1 (Hs00230917_m1), NFE2L2 (Hs00975960_m1), and KEAP1 (Hs00202227_m1) Gene Expression Assays (Applied Biosystems, Foster City, CA, USA). Reactions were done in triplicates, and average *C*_*T*_ values were used to calculate “fold-induction” over vehicle-treated control using Comparative *C*_*T*_ method. ACTB was used as the internal control gene.

### 2.5. Luciferase Reporter Assay

Luciferase reporter assays were carried out as described in our previous study [16]. Briefly, cells grown to 90% confluence in 24-well plates were cotransfected in triplicates with 250 ng of enhancer-luciferase reporter plasmid and 25 ng of pGL4.74[hRluc/TK] internal control plasmid, using Lipofectamine 2000 (Invitrogen, Carlsbad, CA, USA). Six hours after transfection, the medium was replaced with fresh one containing 10 *μ*M ZnPP or same amount of DMSO (vehicle). At 30–48 h after-transfection, the growth medium was removed, and the cells were rinsed twice with ice-cold phosphate buffered saline and were lysed by shaking for 15 min at 25°C with 100 *μ*L of Passive Lysis Reagent (Promega, Madison, WI, USA). Aliquots of 20 *μ*L of the cell lysates were assayed for firefly and renilla luciferase activities using a 20/20 Luminometer (Turner Biosystems, Sunnyvale, CA, USA) and Dual-Luciferase Reporter Assay System (Promega, Madison, WI, USA). The results were expressed as Relative Luciferase Activity (a ratio of the activities of firefly luciferase/renilla luciferase). 

### 2.6. Measurement of Cell Survival

Cells were seeded in triplicates at 0.5–1 × 10^4^ cells/well in 48-well plate in complete medium. At about 25% confluence, cells were treated with various concentrations of ZnPP or vehicle (DMSO) for 48 h. Cell survival was determined using CellTiter 96 Nonradioactive Cell Proliferation Assay (Promega, Madison, WI, USA) according to the protocol provided by the manufacturer. The color developed was measured at 490 nm. 

### 2.7. Western Blot Analysis

PC-3 cells grown to 80% confluence in T25 flasks were treated with 10 *μ*M ZnPP or vehicle for various time intervals up to 48 h. Cells were lysed with 0.5 mL of 1X Laemmli sample buffer containing 1% Halt protease inhibitor and phosphatase inhibitor cocktails (Thermo Scientific, Rockford, IL, USA), sonicated for 2 × 15 sec, and centrifuged at 10,000 rpm for 15 min at 4°C. Aliquots of 50 *μ*g protein extract were analyzed on 10% SDS-polyacrylamide gel and transferred to PVDF membranes. The blots were analyzed by western blot according to the procedure provided by WesternDot 625 kit (Invitrogen, Carlsbad, CA, USA). Briefly, the blots were incubated in 8 mL of Blocking Buffer in a small plastic dish for 1 h at room temperature with gentle agitation. Then they were incubated with the diluted primary antibody (1 : 1000 dilution) at 4°C overnight. After washing 3 times with 50 mL of 1X Wash Buffer, 5 min each, blots were incubated with 8 mL of Biotin-XX-Goat anti-rabbit antibody (1 : 2000 dilution) in Blocking Buffer for 1 h. They were washed 3 times with 50 mL of 1X Wash Buffer for 5 min each, and then incubated with 8 mL of Qdot 625 streptavidin conjugate (1 : 2000 dilution) in Blocking Buffer for 60 min at room temperature. Finally, the blots were washed 3 times with 50 mL of 1X Wash Buffer for 5 min each, and once with 20 mL of ultrapure water. The wet blots were placed on a UV trans-illuminator and pictures were taken with a Polaroid camera and orange filter. 

### 2.8. Data Analysis

Data points shown represent mean ± standard error. Statistically significant differences between data points of two groups were determined by Student's *t*-test. By convention, a *P* value of <0.05 was considered statistically significant.

## 3. Results

**Figure 1:**
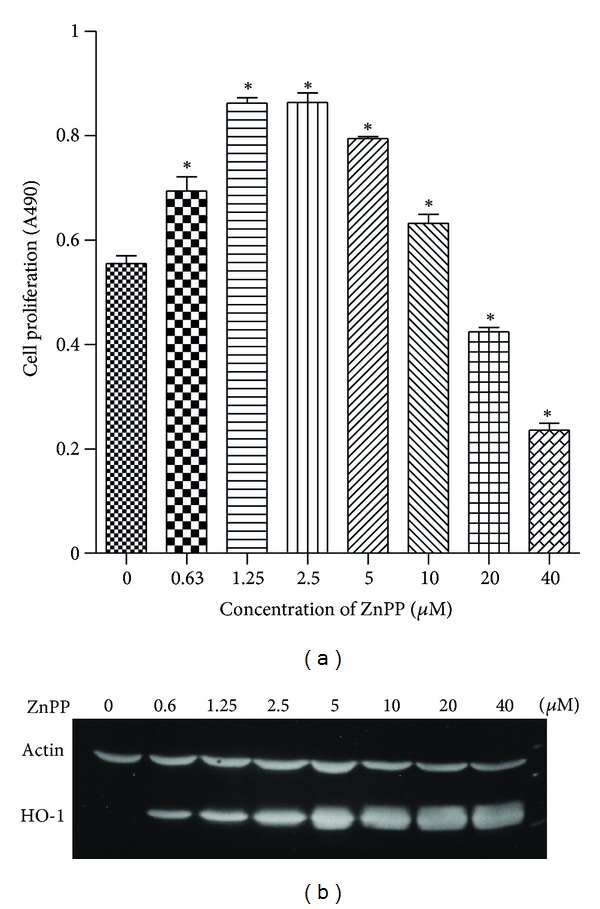
Effect of ZnPP on cell proliferation and HO-1 expression of PC-3 cells. (a) PC-3 cells were treated with various concentrations of ZnPP for 48 h, and number of live cells was estimated by MTS cell proliferation assay as described in Section 2. Results were expressed as Absorbance at 490 nm (mean ± S.E.). *N* = 3; *P < 0.05 compared with untreated control. (b) PC-3 cells were treated with various concentrations of ZnPP for 24 h, and HO-1 expression was determined by western blot analysis as described in Section 2 using specific antibodies against *β*-actin and HO-1. Immunoreactive protein bands detected by WesternDot 625 appeared as fluorescent bands.

**Figure 2:**
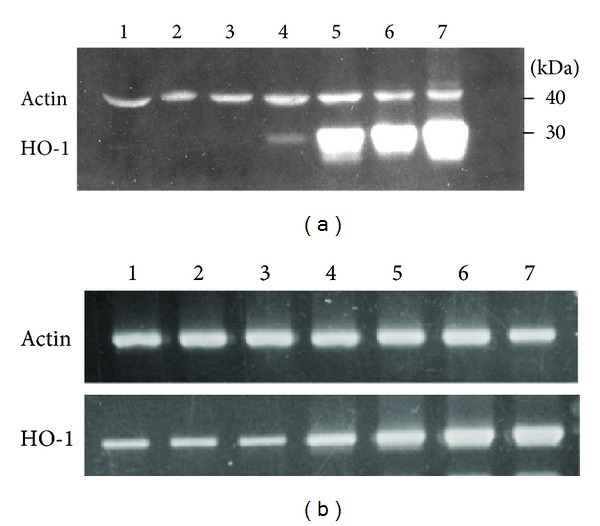
Induction of HO-1 by ZnPP in PC-3 for various lengths of time. PC-3 cells were treated with 10 *μ*M ZnPP for 0.5, 1, 4, 16, 24, and 48 h (lanes 2, 3, 4, 5, 6, and 7, resp.). Control cells were treated with equal amount of DMSO for 48 h (lane 1). (a) Western blot analysis was done as described in Section 2, using specific antibodies against *β*-actin and HO-1. Immunoreactive protein bands detected by WesternDot 625 appeared as fluorescent bands. (b) RT-PCR analysis using gene-specific primers. PCR fragments were visualized by ethidium bromide staining.

ZnPP is relatively nontoxic to PC-3 cells. In fact, it induced significant cell proliferation at a concentration of 0.6–10 *μ*M, and only suppressed cell growth above 10 *μ*M (Figure 1(a)). Therefore, 10 *μ*M ZnPP was used for all subsequent experiments. Basal expression level of HO-1 protein in PC-3 was undetectable. ZnPP induced HO-1 protein expression in a dose-dependent manner, with the highest induction level at 10 *μ*M (Figure 1(b)). For a time course study, incubation of the cells with 10 *μ*M ZnPP for 4 h showed only a slight induction of HO-1 protein, but the induction was high after 16 h and was maintained through 48 h of incubation (Figure 2(a)). The HO-1 mRNA level as determined by RT-PCR also showed similar profile (Figure 2(b)).

**Figure 3:**
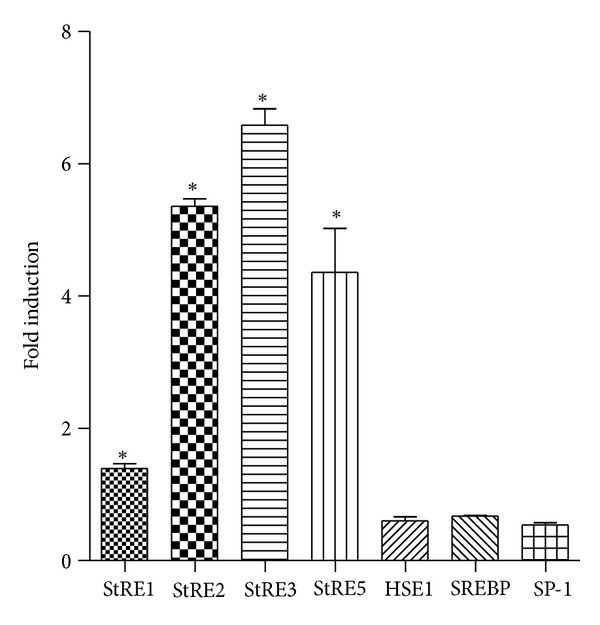
Effect of ZnPP on StRE1, StRE2, StRE3, StRE5, HSE, SREBP, and SP1 elements of human HO-1 promoter in PC-3 cells. PC-3 cells were transfected with enhancer-luciferase reporter plasmid harboring one of these elements, treated with 10 *μ*M ZnPP for 48 h, and luciferase activities were determined as described in Section 2. Results were expressed as “Fold Induction” over vehicle (DMSO)-treated control of the corresponding responsive element (mean ± S.E.). *N* = 3; *P < 0.05 compared with vehicle-treated control of the corresponding responsive element.

Of all the known responsive elements in the HO-1 promoter, ZnPP activated mainly the ARE-like elements (StREs). As shown in Figure 3, StRE3 showed the highest (6.6-fold) induction level by ZnPP, although these elements had different basal expression levels of relative luciferase activities due to different copy number of the response elements present in the luciferase-reporter constructs. ZnPP did not activate the HSE, SREBP, and SP1 elements (Figure 3).

**Figure 4:**
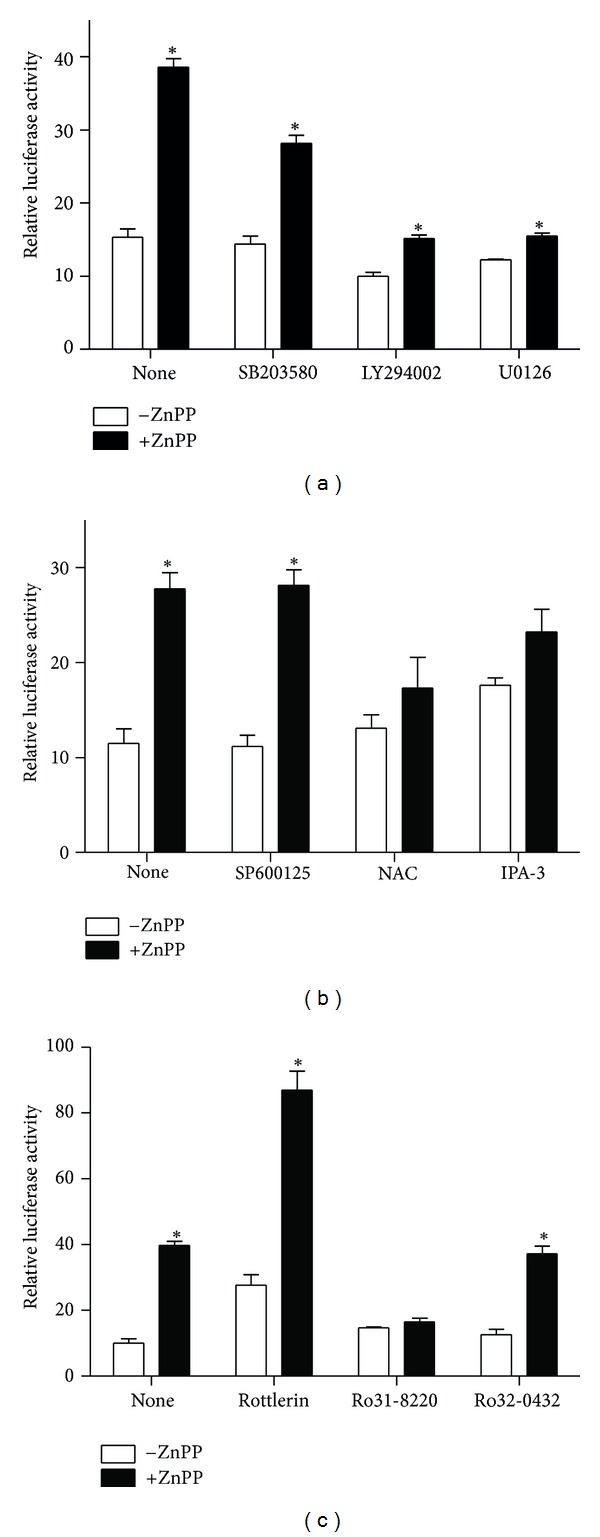
Effect of protein kinase inhibitors and antioxidant on ZnPP-activation of StRE3 element of human HO-1 promoter in PC-3 cells. PC-3 cells were transfected with StRE3-pGL3 luciferase reporter plasmid, pretreated with 3 *μ*M SB203580, 5 *μ*M LY294002, 10 *μ*M U0126, 3 *μ*M SP600125, 500 *μ*M NAC, 10 *μ*M IPA-3, 2 *μ*M rottlerin, 5 *μ*M Ro 31-8220, or 5 *μ*M Ro 32-0432 for 2 h, and then treated with 10 *μ*M ZnPP for 24 h, and luciferase activities were determined as described in Section 2. Results were expressed as “relative luciferase activity (ratio of activities of firefly luciferase/renilla luciferase)” (mean ± S.E.). *N* = 3; *P < 0.05 compared with the untreated control.

A number of protein kinases are known to be involved with the activation of antioxidant response element. To investigate the effect of various protein kinase inhibitors and antioxidant on the activation of StRE by ZnPP, cells transfected with StRE3-pGL3 were pretreated for 2 h with SB203580 (p38-MAPK inhibitor), LY294002 (phosphatidylinositol 3-kinase inhibitor), U0126 (MEK inhibitor), SP600125 (JNK inhibitor), IPA-3 (p21-Activated Kinase Inhibitor III), NAC (antioxidant), rottlerin (PKC-*δ* inhibitor), Ro 31-8220 (pan PKC inhibitor), or Ro 32-0432 (PKC-*α* inhibitor) prior to treatment with ZnPP for 24 h. As shown in Figure 4, SP600125 and Ro 32-0432 had little effect on the activation of StRE3 by ZnPP. SB203580, NAC, and IPA-3 reduced the activation of StRE3 by ZnPP to 72.9%, 62.4%, and 83.6%, respectively, of the level by ZnPP alone. However, LY294002, U0126, and Ro 31-8220 attenuated the activation to 39.4%, 40.2%, and 41.5%, respectively, of the ZnPP-alone control. On the other hand, rottlerin activated StRE3 element by itself and had a synergistic effect with ZnPP (2-fold over the level by ZnPP alone).

**Figure 5:**
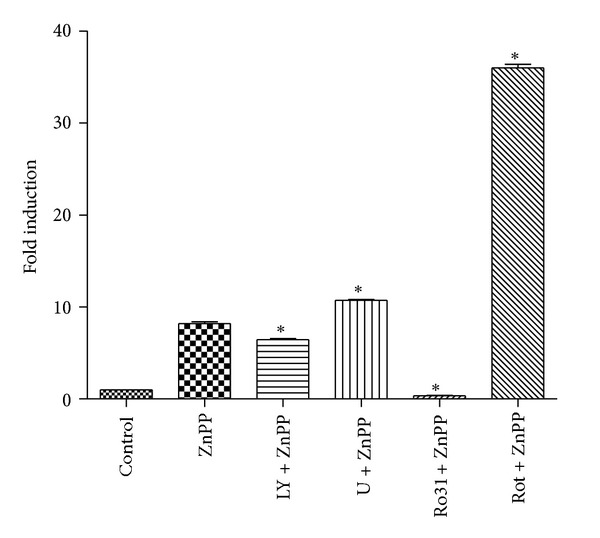
Real-time PCR analyses of HO-1 mRNA levels in PC-3 cells treated with ZnPP in the presence of various kinase inhibitors. PC-3 cells were pretreated with 5 *μ*M LY294002, 10 *μ*M U0126, 5 *μ*M Ro 31-8220, or 2 *μ*M rottlerin for 1 h, and then treated with 10 *μ*M ZnPP for 3 h, and relative HO-1 mRNA levels were determined by real-time PCR as described in Section 2. Results were expressed as “Fold Induction” over vehicle (DMSO-) treated control. *N* = 3; *P < 0.05 compared with ZnPP alone.

**Figure 6:**
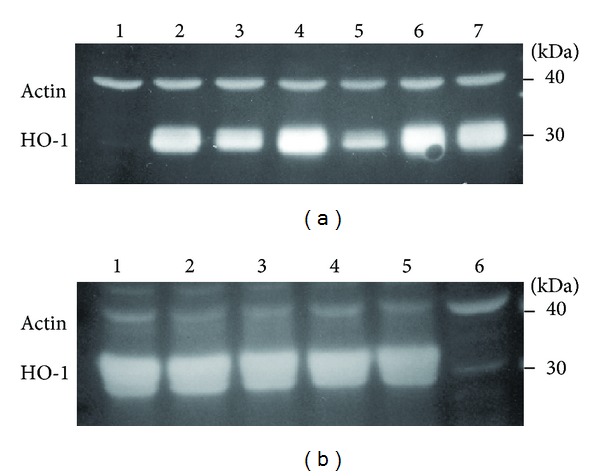
Western blot analysis of HO-1 protein induced by ZnPP in PC-3 in the presence of various kinase inhibitors or antioxidant. (a) PC-3 cells were pretreated with nothing (lane 2), 5 *μ*M LY294002 (lane 3), 10 *μ*M U0126 (lane 4), 5 *μ*M Ro 31-8220 (lane 5), 2 *μ*M rottlerin (lane 6), or 5 *μ*M Ro 32-0432 (lane 7) for 2 h, and then treated with 10 *μ*M ZnPP for 24 h. Control cells were treated with equal amount of DMSO for 24 h (lane 1). Total proteins (50 *μ*g) were analyzed by western blot as described in Section 2, using specific antibodies against *β*-actin and HO-1. Immunoreactive protein bands detected by WesternDot 625 appeared as fluorescent bands. (b) PC-3 cells were pretreated with 5 *μ*M myristoylated PKC-*θ* pseudosubstrate (lane 1), 5 *μ*M myristoylated PKC-*ζ* pseudosubstrate (lane 2), 5 *μ*M myristoylated PKC-*η* pseudosubstrate (lane 3), 5 *μ*M PKC-**β** inhibitor (lane 4), or nothing (lane 5) for 2 h, and then treated with 10 *μ*M ZnPP for 24 h. Control cells were treated with equal amount of DMSO for 24 h (lane 6). Western blot analysis was done as described above.

To confirm the effect of LY294002, U0126, Ro 31-8220, and rottlerin on ZnPP-activation of StRE3 element, HO-1 mRNA levels were determined by real-time PCR and protein levels by western blot analyses. For real-time PCR, the relative levels of HO-1 mRNA were determined in cells pretreated with LY294002, U0126, Ro 31-8220, or rottlerin for 1 h prior to ZnPP treatment for 3 h. The results showed that LY294002 and U0126 did not attenuate ZnPP-induction of HO-1 mRNA, but Ro 31-8220 completely suppressed the effect of ZnPP (Figure 5). On the other hand, real-time PCR also confirmed the synergistic effect of rottlerin with ZnPP; ZnPP alone upregulated HO-1 by 8.2-fold over vehicle-treated control and rottlerin plus ZnPP upregulated HO-1 by 36.0-fold (Figure 5). Western blot analyses basically confirmed the results of real-time PCR. Ro 31-8220 significantly suppressed the upregulation of HO-1 protein by ZnPP, while LY294002, U0126, and Ro 32-0432 had little effect on ZnPP-induction of HO-1 (Figure 6(a)). However, the synergistic effect of rottlerin with ZnPP was not evident due to the high level of upregulation of HO-1 by ZnPP alone. Western blot analysis also showed that SB203580 and IPA-3 had no effect on ZnPP-induction of HO-1 expression (data not shown).

Since Ro 31-8220 is a pan PKC inhibitor, this suggests that PKC may be involved in ZnPP-upregulation of HO-1. There are many PKC isoforms, but the involvement of PKC-*α* can be excluded by the lack of suppression of Ro 32-0432 (PKC-*α* inhibitor) on ZnPP-upregulation of HO-1 protein (Figure 6(a), lane 7). To determine if other PKC isoforms may be involved in ZnPP-activation of HO-1, PC-3 cells were treated with myristoylated pseudosubstrates of PKC-*θ*, PKC-*ζ* and PKC-*η*, or PKC-*β* inhibitor prior to ZnPP treatment. Western blot analysis showed no effect of these inhibitors on HO-1 protein level (Figure 6(b)). Hence, involvement of PKC-*θ*, PKC-*ζ* and PKC-*η*, and PKC-*β* can also be excluded. 

**Figure 7:**
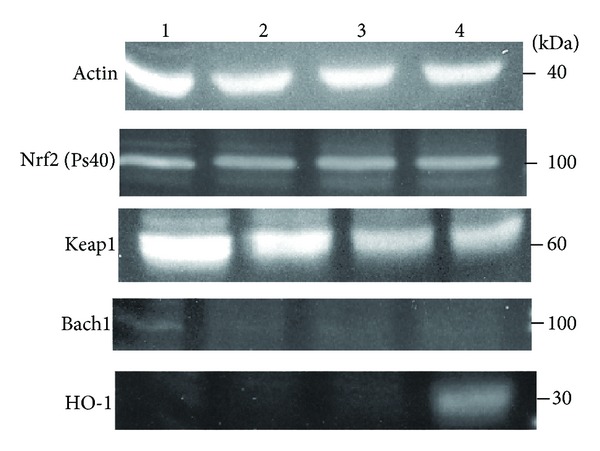
Effect of ZnPP on the level of phospho-Nrf2(pS40), Keap1, Bach1, and HO-1 in PC-3. PC-3 cells were treated with 10 *μ*M ZnPP for 2 h (lane 2), 4 h (lane 3), and 24 h (lane 4). Vehicle-treated cells were served as control (lane 1). Total proteins (50 *μ*g) were analyzed by western blot as described in Section 2, using specific antibodies against *β*-actin, phospho-Nrf2(pS40), Keap1, Bach1, and HO-1. Immunoreactive protein bands detected by WesternDot 625 appeared as fluorescent bands.

Since Bach1, Nrf2 and Keap1 proteins have been shown to interact with antioxidant response element, the effect of ZnPP on the mRNA and protein levels of these proteins were investigated by real-time PCR and Western blot analyses. Real-time PCR analyses showed that there were no drastic changes in the expression of BACH1, NFE2L2 (NRF2), and KEAP1 genes when PC-3 cells were treated with ZnPP. The expression levels of BACH1, NFE2L2, and KEAP1 in cells treated with 10 *μ*M ZnPP for 3 h were 73.5, 81.8, and 98.8%, respectively, of those in control cells. Western blot analysis showed no change in phospho-Nrf2(pS40) protein but a gradual decrease in Keap1 protein in cells treated with ZnPP for 2–24 h (Figure 7). Only a faint band of Bach1 protein was detected in PC-3 control cells, but none at all in cells treated with ZnPP, despite antibodies from two companies were used. To investigate the effect of ZnPP on the expression of other antioxidant responsive genes, the expression levels of NQO1 and GSTP1 in cells treated with 10 *μ*M ZnPP for 3 h were determined by real-time PCR. The results showed that the expression levels of NQO1 and GSTP1 were 89.8 and 77.7%, respectively, of those in control cells. 

**Figure 8:**
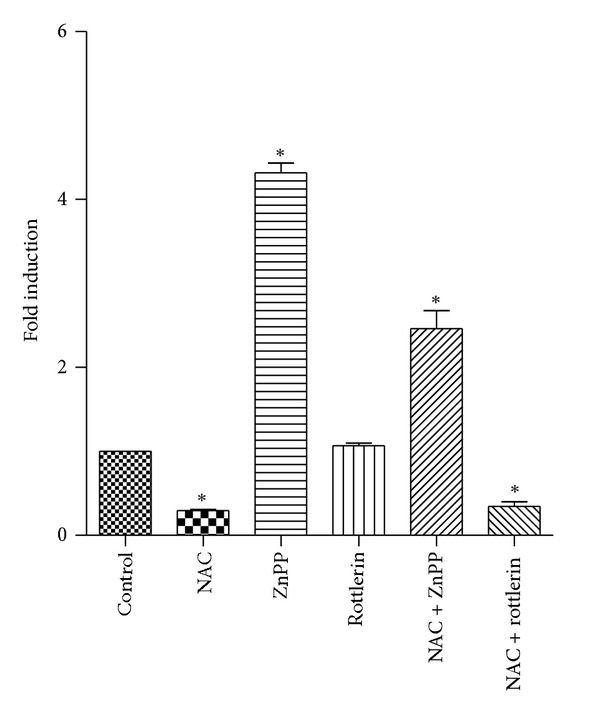
Effect of NAC on ZnPP- or rottlerin-induction of HO-1. PC-3 cells were treated with nothing (control); 10 mM NAC (5 h); 10 *μ*M ZnPP (5 h); 2 *μ*M rottlerin (5 h); 10 mM NAC (1 h pretreatment) plus 10 *μ*M ZnPP (5 h); or 10 mM NAC (1 h pretreatment) plus 2 *μ*M rottlerin (5 h). Relative HO-1mRNA levels were determined by real-time PCR as described in Section 2. Results were expressed as “Fold Induction” over control. *N* = 3; *P < 0.05 compared with control.

To determine if rottlerin and ZnPP can act as prooxidant, cells were pretreated with 10 mM NAC prior to incubation with 10 *μ*M ZnPP or 2 *μ*M rottlerin, and relative levels of HO-1 mRNA were determined by real-time PCR. The results showed that NAC, even at 10 mM, reduced ZnPP-induction of HO-1 expression by only 43% (Figure 8). Rottlerin by itself did not induce HO-1 mRNA expression after 5 h incubation (Figure 8), and only a faint band of HO-1 protein was detected on western blot after 24 h incubation with rottlerin (data not shown). Therefore, the effect of NAC on rottlerin was not determined.

## 4. Discussion

In this study, we demonstrated that ZnPP upregulated HO-1 expression in PC-3 cells in a dose-dependent manner, and that upregulation was done mainly through activation of the ARE-like response elements (StREs) of HO-1 promoter. Although similar response elements are present in the promoter of other antioxidant responsive genes, such as NQO1 and GSTP1 [17, 18], real-time PCR analysis showed no upregulation of these genes by ZnPP. It should be noted that the induction of HO-1 protein is much stronger than that of HO-1 mRNA, suggesting ZnPP may also affect other pathways that stabilize the HO-1 protein level. However, no known protease inhibitor activity of ZnPP has been reported. We also demonstrated that preincubation of the cells with Ro 31-8220, a pan inhibitor of PKC, abrogated ZnPP-induction of HO-1 expression, suggesting that activation of HO-1 gene by ZnPP may involve PKC. 

It is intriguing to note that induction of HO-1 expression in PC-3 cells by hemin inhibited cell proliferation [4], whereas our results showed that although ZnPP also induced HO-1 expression in PC-3 cells, proliferation was not inhibited until ZnPP concentration exceeded 10 *μ*M (Figure 1(a)). This may be due to the fact that ZnPP is also a well-known HO-1 inhibitor. HO-1 enzymatic activity may be required for the inhibition of cell proliferation. When ZnPP level was increased to above 10 *μ*M, HO-1 level finally overwhelmed the inhibitory effect of ZnPP. 

The ability of ZnPP to activate the ARE-like response elements (StREs) of HO-1 promoter suggests that ZnPP upregulates HO-1expression via the Nrf2-ARE signaling pathway. The level of Nrf2 is regulated by Keap1. The binding of Nrf2 by Keap1 results in the ubiquitination and degradation of Nrf2. Phosphorylation of Nrf2 and/or modification of cysteine residues of Keap1 results in decreased Nrf2 ubiquitination and degradation, and hence activation of the ARE signaling pathway [19]. Phosphorylation of Nrf2 at Ser^40^ residue by PKC results in dissociation of Nrf2 from the Nrf2-Keap1 complex, translocation of Nrf2 into nucleus, and activation of ARE-like element of genes responsive to oxidative stress [20, 21]. Our results showed no change in phospho-Nrf2 (pS40) level in ZnPP-treated cells as compared to control (Figure 7). However, our results did show a progressive decrease in Keap1 level in ZnPP-treated cells (Figure 7). The significance of the decrease in Keap1 level remains to be studied. On the other hand, ARE is known to be bound and repressed by a transcription factor called Bach1. Inactivation of the repressor Bach1 will also lead to the activation of ARE [22]. However, Bach1 is unlikely involved in the upregulation of HO-1 by ZnPP for two reasons. First, basal expression level of Bach1 in PC-3 cells is very low; it was barely detectable in control cells by western blot (Figure 7), and no downregulation of Bach1 mRNA induced by ZnPP was detected by real-time PCR. Second, preincubation of the cells with 10 mM NAC prior to ZnPP treatment did not completely abrogate the ZnPP-induction of HO-1 (Figure 8). This suggests that ZnPP does not act as a prooxidant that would inactivate Bach1.

Since upregulation of HO-1 by ZnPP can be suppressed by Ro 31-8220, ZnPP-induction may be mediated by PKC. The involvement of PKC-*δ* in the upregulation of HO-1 by many phyto-chemicals has been demonstrated, as rottlerin and PKC-*δ* small interfering RNA were able to attenuate HO-1 induction by these compounds [23–27]. It should be noted that although rottlerin is not an efficient PKC-*δ* inhibitor [28], the involvement of PKC-*δ* was confirmed by the use of PKC-*δ* small interfering RNA in these studies. On the other hand, involvement of PKC-*ζ* [29] and yet unidentified atypical PKC [30] has also been demonstrated. Our results showed that preincubation of PC-3 cells with Ro 32-0432, PKC-*β* inhibitor, rottlerin, and pseudosubstrates of PKC-*ζ*, PKC-*θ*, and PKC-*η* did not suppress ZnPP-induction of HO-1 expression, and hence the involvement of *α*, *β*, *δ*, *η*, *θ*, and *ζ* isoforms of PKC can be ruled out. PC-3 cells are known to express PKC *α*, *δ*, *ε*, *η*, and *μ* isoforms as determined by nuclease protection assay [31] and *α*, *ε*, *ζ*, and *ι* as determined by western blot [32]. Involvement of PKC *ε*, *ι*, and *μ* isoforms has not been tested in this study, because specific inhibitors for these isoforms are not commercially available. The PKC isoform involved in ZnPP-induction of HO-1 remains to be determined. 

Our results showed that rottlerin did not attenuate ZnPP-induction of HO-1. Instead, rottlerin had a synergistic effect with ZnPP, and this was confirmed at the mRNA level (Figure 5). There is at least one report showing that rottlerin was able to upregulate HO-1 through reactive oxygen species (ROS) dependent and PKC-*δ*-independent pathway in human colon cancer HT29 cells, as its induction was abrogated by antioxidant NAC but not by suppression of PKC-*δ* expression by small interfering RNA technology [33]. In PC-3 cells, rottlerin did not induce HO-1 to any significant level by itself, but had a synergistic effect with ZnPP. 

Ro 31-8220, a pan inhibitor of PKC, has been reported to induce apoptosis independent of PKC activity [34]. However, our results showed that Ro 31-8220 was able to suppress ZnPP-induction of HO-1 mRNA after only 3 h of incubation (Figure 5). Furthermore, PC-3 cells treated with 5 *μ*M Ro 31-8220 for 24 h retained 93% viability (data not shown). These suggest that the suppression of ZnPP-induction of HO-1 by Ro 31-8220 was not due to induction of apoptosis. On the other hand, Ro 31-8220 has been shown to inhibit other kinases, such as MAPKAP kinase-1*β* (also known as Rsk-2) and p70 S6 kinase [35]. Furthermore, Ro 31-8220 has been shown to activate JNK1 [36]. Involvement of these kinases in ZnPP-activation has not been ruled out.

In conclusion, ZnPP upregulates HO-1 in PC-3 cells via the activation of StRE of HO-1 promoter. The pathway through which the StRE is activated remains to be determined.

## Abbreviations

ARE: Antioxidant response element
Bach1: Broad-complex, tramtrack and bric à brac and cap'n'collar homology 1
DMSO: Dimethyl sulfoxide
Egr-1: Early growth response 1
GSTP1: Glutathione S-transferase P1
HO-1: Heme oxygenase-1
HSE: Heat-shock response element
JNK: C-Jun N-terminal kinase
Keap1: Kelch-like ECH-associated protein 1
MAPK: Mitogen-activated protein kinase
MAPKAPK: MAPK-activated protein kinase
MEK: Mitogen-activated protein/extracellular signal-regulated kinase kinase
NAC: N-acetyl cysteine
NQO1: NAD(P)H quinone oxidoreductase 1
Nrf2: Nuclear factor erythroid-derived 2 related factor 2
PKC: Protein kinase C
ROS: Reactive oxygen species
SREBP: Sterol regulatory element binding transcription factor 1
SP1: SP1 transcription factor
StRE: Stress response element
ZnPP: Zinc protoporphyrin IX.

## References

[1] Bauer M, Huse K, Settmacher U, Claus RA (2008). The heme oxygenase-carbon monoxide system: regulation and role in stress response and organ failure. *Intensive Care Medicine*.

[2] Jozkowicz A, Was H, Dulak J (2007). Heme oxygenase-1 in tumors: is it a false friend?. *Antioxidants and Redox Signaling*.

[3] Maines MD, Abrahamsson PA (1996). Expression of heme oxygenase-1 (HSP32) in human prostate: normal, hyperplastic, and tumor tissue distribution. *Urology*.

[4] Gueron G, De Siervi A, Ferrando M (2009). Critical role of endogenous heme oxygenase 1 as a tuner of the invasive potential of prostate cancer cells. *Molecular Cancer Research*.

[5] Sacca P, Meiss R, Casas G (2007). Nuclear translocation of haeme oxygenase-1 is associated to prostate cancer. *British Journal of Cancer*.

[6] Alam J, Cook JL (2003). Transcriptional regulation of the heme oxygenase-1 gene via the stress response element pathway. *Current Pharmaceutical Design*.

[7] Alam J, Cook JL (2007). How many transcription factors does it take to turn on the heme oxygenase-1 gene?. *American Journal of Respiratory Cell and Molecular Biology*.

[8] Okinaga S, Takahashi K, Takeda K (1996). Regulation of human heme oxygenase-1 gene expression under thermal stress. *Blood*.

[9] Kallin A, Johannessen LE, Cani PD (2007). SREBP-1 regulates the expression of heme oxygenase 1 and the phosphatidylinositol-3 kinase regulatory subunit p55*γ*. *Journal of Lipid Research*.

[10] Deshane J, Kim J, Bolisetty S, Hock TD, Hill-Kapturczak N, Agarwal A (2010). Sp1 regulates chromatin looping between an intronic enhancer and distal promoter of the human heme oxygenase-1 gene in renal cells. *Journal of Biological Chemistry*.

[11] Yang G, Nguyen X, Ou J, Rekulapelli P, Stevenson DK, Dennery PA (2001). Unique effects of zinc protoporphyrin on HO-1 induction and apoptosis. *Blood*.

[12] Hirai K, Sasahira T, Ohmori H, Fujii K, Kuniyasu H (2007). Inhibition of heme oxygenase-1 by zinc protoporphyrin IX reduces tumor growth of LL/2 lung cancer in C57BL mice. *International Journal of Cancer*.

[13] Fang J, Greish K, Qin H (2012). HSP32 (HO-1) inhibitor, copoly(styrene-maleic acid)-zinc protoporphyrin IX, a water-soluble micelle as anticancer agent: *in vitro* and *in vivo* anticancer effect. *European Journal of Pharmaceutics and Biopharmaceutics*.

[14] Hsieh CH, Jeng JCY, Hsieh MW (2011). Involvement of the p38 pathway in the differential induction of heme oxygenase-1 by statins in Neuro-2A cells exposed to lipopolysaccharide. *Drug and Chemical Toxicology*.

[15] Kwok SCM, Samuel SP, Handal J (2012). Atorvastatin activates heme oxygenase-1 at the stress response elements. *Journal of Cellular and Molecular Medicine*.

[16] Kwok SCM, Daskal I (2008). Brefeldin A activates CHOP promoter at the AARE, ERSE and AP-1 elements. *Molecular and Cellular Biochemistry*.

[17] Jaiswal AK (2000). Regulation of genes encoding NAD(P)H:quinone oxidoreductases. *Free Radical Biology and Medicine*.

[18] Nishinaka T, Ichijo Y, Ito M (2007). Curcumin activates human glutathione S-transferase P1 expression through antioxidant response element. *Toxicology Letters*.

[19] Giudice A, Arra C, Turco MC (2010). Review of molecular mechanisms involved in the activation of the Nrf2-ARE signaling pathway by chemopreventive agents. *Methods in Molecular Biology*.

[20] Huang HC, Nguyen T, Pickett CB (2002). Phosphorylation of Nrf2 at Ser-40 by protein kinase C regulates antioxidant response element-mediated transcription. *Journal of Biological Chemistry*.

[21] Bloom DA, Jaiswal AK (2003). Phosphorylation of Nrf2 at Ser40 by Protein Kinase C in Response to Antioxidants Leads to the Release of Nrf2 from INrf2, but Is Not Required for Nrf2 Stabilization/Accumulation in the Nucleus and Transcriptional Activation of Antioxidant Response Element-mediated NAD(P)H:Quinone Oxidoreductase-1 Gene Expression. *Journal of Biological Chemistry*.

[22] Reichard JF, Motz GT, Puga A (2007). Heme oxygenase-1 induction by NRF2 requires inactivation of the transcriptional repressor BACH1. *Nucleic Acids Research*.

[23] Rushworth SA, Ogborne RM, Charalambos CA, O’Connell MA (2006). Role of protein kinase C *δ* in curcumin-induced antioxidant response element-mediated gene expression in human monocytes. *Biochemical and Biophysical Research Communications*.

[24] Kim BC, Jeon WK, Hong HY (2007). The anti-inflammatory activity of Phellinus linteus (Berk. & M.A. Curt.) is mediated through the PKC*δ*/Nrf2/ARE signaling to up-regulation of heme oxygenase-1. *Journal of Ethnopharmacology*.

[25] Ogborne RM, Rushworth SA, O’Connell MA (2008). Epigallocatechin activates haem oxygenase-1 expression via protein kinase C*δ* and Nrf2. *Biochemical and Biophysical Research Communications*.

[26] Zhang H, Forman HJ (2008). Acrolein induces heme oxygenase-1 through PKC-*δ* and PI3K in human bronchial epithelial cells. *American Journal of Respiratory Cell and Molecular Biology*.

[27] Lee SE, Jeong SI, Yang H (2011). Fisetin induces Nrf2-mediated HO-1 expression through PKC-*δ* and p38 in human umbilical vein endothelial cells. *Journal of Cellular Biochemistry*.

[28] Soltoff SP (2007). Rottlerin: an inappropriate and ineffective inhibitor of PKC*δ*. *Trends in Pharmacological Sciences*.

[29] Rojo AI, Salina M, Salazar M (2006). Regulation of heme oxygenase-1 gene expression through the phosphatidylinositol 3-kinase/PKC-*ζ* pathway and Sp1. *Free Radical Biology and Medicine*.

[30] Numazawa S, Ishikawa M, Yoshida A, Tanaka S, Yoshida T (2003). Atypical protein kinase C mediates activation of NF-E2-related factor 2 in response to oxidative stress. *American Journal of Physiology-Cell Physiology*.

[31] Powell CT, Brittis NJ, Stec D, Hug H, Heston WDW, Fair WR (1996). Persistent membrane translocation of protein kinase C *α* during 12-O- tetradecanoylphorbol-13-acetate-induced apoptosis of LNCaP human prostate cancer cells. *Cell Growth and Differentiation*.

[32] Stewart JR, O’Brian CA (2004). Resveratrol antagonizes EGFR-dependent Erk1/2 activation in human androgen-independent prostate cancer cells with associated isozyme-selective PKC*α* inhibition. *Investigational New Drugs*.

[33] Park EJ, Lim JH, Nam SI, Park JW, Kwon TK (2010). Rottlerin induces heme oxygenase-1 (HO-1) up-regulation through reactive oxygen species (ROS) dependent and PKC *δ*-independent pathway in human colon cancer HT29 cells. *Biochimie*.

[34] Han Z, Pantazis P, Lange TS, Wyche JH, Hendrickson EA (2000). The staurosporine analog, Ro-31-8220, induces apoptosis independently of its ability to inhibit protein kinase C. *Cell Death and Differentiation*.

[35] Alessi DR (1997). The protein kinase c inhibitors Ro 318220 and GF 109203X are equally potent inhibitors of MAPKAP kinase-1*β* (Rsk-2) and p70 S6 kinase. *FEBS Letters*.

[36] Beltman J, McCormick F, Cook SJ (1996). The selective protein kinase C inhibitor, Ro-31-8220, inhibits mitogen- activated protein kinase phosphatase-1 (MKP-1) expression, induces c-Jun expression, and activates Jun N-terminal kinase. *Journal of Biological Chemistry*.

